# In vitro antifungal activity of *Cinnamomum zeylanicum* bark and leaf essential oils against *Candida albicans* and *Candida auris*

**DOI:** 10.1007/s00253-020-10829-z

**Published:** 2020-09-03

**Authors:** Hoang N. H. Tran, Lee Graham, Emmanuel C. Adukwu

**Affiliations:** 1 Faculty of Life Sciences, School of Physiology, Pharmacology and Neuroscience, University of Bristol, Bristol, BS8 1TH UK; 2 Centre for Research in Biosciences, University of the West of England, Coldharbour Lane, Bristol, BS16 1QY UK

**Keywords:** *Candida* infections, Antifungal, Antihaemolytic, Essential oil, Cinnamon

## Abstract

*Candida* infections are a significant source of patient morbidity and mortality. *Candida albicans* is the most common pathogen causing *Candida* infections. *Candida auris* is a newly described pathogen that is associated with multi-drug-resistant candidiasis and candidaemia in humans. The antifungal effects of various essential oils and plant compounds have been demonstrated against human pathogenic fungi. In this study, the effect of cinnamon leaf and bark essential oils (CEOs) was determined against both *C. albicans* and *C. auris*. The disc diffusion (direct and vapour) and broth microdilution method was used to determine antifungal activity of the EOs against selected strains (*C. albicans* ATCC 10231, *C. albicans* ATCC 2091 and *C. auris* NCPF 8971) whilst the mode of action and haemolysin activity of the CEOs were determined using electron microscopy and light microscopy. Direct and vapour diffusion assays showed greater inhibitory activity of bark CEO in comparison with leaf CEO. The minimum inhibitory concentrations (MICs) and minimum fungicidal concentrations (MFCs) of bark CEO for all tested strains was below 0.03% (*v*/*v*), which was lower than the MICs of the leaf CEO (0.06–0.13%, *v*/*v*) dependent on the strain and the MFCs at 0.25% (*v*/*v*). In the morphological interference assays, damage to the cell membrane was observed and both CEOs inhibited hyphae formation. The haemolysin production assay showed that CEOs can reduce the haemolytic activity in the tested *C. albicans* and *C. auris* strains. At low concentrations, CEOs have potent antifungal and antihaemolytic activities in vitro against *C. albicans* and *C. auris*.

**Key points**

*• Essential oils from Cinnamomum zeylanicum Blume bark and leaf (CBEO and CLEO) demonstrated fungicidal properties at very low concentrations.*

*• The antifungal activity of CBEO was greater than that of CLEO consistent with other recent published literature.*

*• The mode of action of CBEO and CLEO was damage to the membrane of C. albicans and C. auris.*

*• Both CBEO and CLEO inhibited the formation of hyphae and reduced haemolysin production in C. albicans and C. auris.*

**Figure:**
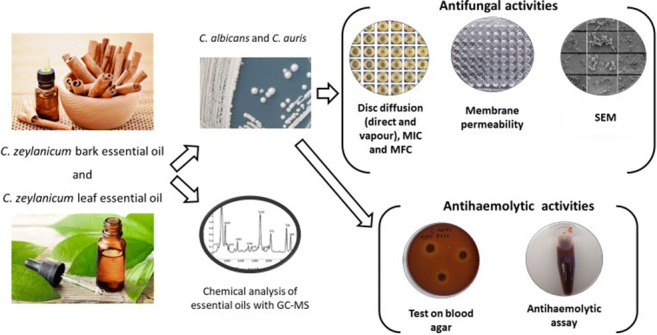

## Introduction

Fungal infections are common diseases of the natural world. In humans, fungal infections occur when the immune system is unable to deal with a considerable amount of trespassing fungi or yeast invading an area of the body. There are several classes of fungal infections, and of these, *Candida* infections are regarded as a significant cause of patient morbidity and mortality (Sardi et al. 2013). *Candida* infections are caused by commensal fungi *Candida* spp*.*, especially *Candida albicans*, which resides on the skin, mucosa and gastrointestinal tract of 30 to 50% of healthy adults at any given time, with everyone being colonized at some point in their lifetime (Brown and Netea 2007). *Candida auris* is a newly described opportunistic pathogen, which grows as yeast. *C. auris* is known to be resistant to several classes of antifungal drugs including the first-line antifungal, fluconazole, and exhibits variable susceptibility to other azoles, amphotericin B, and echinocandins (Chowdhary et al. 2016). Additionally, *C. auris* has a potential for person-to-person transmission, challenging clinicians and infection control teams (Ben-Ami et al. 2017).

Essential oils (EOs) are considered plant-originated complex liquid mixtures of volatile and low molecular weight substances (Amorati et al. 2013; Raut and Karuppayil 2014). Recently, natural products extracted from traditional medicine have contributed to the development of innovative modern drugs (Yunes et al. 2005; Almeida et al. 2001). Antifungal activity against various plant and human pathogenic fungi including yeasts have been discovered in numerous EOs (Raut and Karuppayil 2014; Chen et al. 2013; Bajpai et al. 2009; Li et al. 2014). *Cinnamomum zeylanicum* Blume (a synonym of *Cinnamon verum* J. Presl) commonly known as Sri Lanka cinnamon is widely cultivated in India, China, Sri Lanka, and Australia (Prasad et al. 2009). The EOs made from its leaf and bark have been described as having useful antiseptic, immunostimulant, detoxifying, analgesic and antidepressant effects (Thosar et al. 2013; Gautam et al. 2014; Vaseeharan and Thaya 2014; Tworkoski 2002; Topa et al. 2018).

In India, Dalchini (*C. zeylanicum* Blume bark) is often soaked in water and used as mouthwash solution to treat dental caries pathogens. The study of Aneja et al. (2009) showed that Dalchini extract exhibits even much higher antifungal activity than the standard antifungal drug amphotericin-B on oral pathogens including *Saccharomyces cerevisiae* and *C. albicans.* In addition, the extraction of cinnamon leaf and bark demonstrated inhibitory activity against *Fusarium graminearum*, *F. proliferatum*, *Aspergillus fumigatus* and *Trichophyton rubrum* in previous studies (Velluti et al. 2003; Velluti et al. 2004; Khan and Ahmad 2011). In the study by Carvalho et al. (2018), CEOs extracted from *C. zeylanicum* were shown to inhibit some virulence factors in clinical strains of *C. albicans* such as proteinase production, germ tube formation and adhesion to buccal epithelial cells. Moreover, previous studies have reported that cinnamaldehyde and eugenol, which are the main components of bark and leaf cinnamon essential oils, respectively, have demonstrated active in vitro tested against dermatophyte strains isolated from patients with dermatophytosis inhibiting 80% of the dermatophyte strains tested (Gruenwald et al. 2010). However, to our knowledge, there is little data available demonstrating the possibility of utilizing CEOs to treat infections caused by *C. albicans* and no evidence to date of EO activity against *C. auris*.

The focus of this study was to investigate the antifungal activities and the antihaemolytic effects of the oils extracted from leaf and bark of *C. zeylanicum* Blume against *C. albicans* and *C. auris.*

## Methods

### Fungal organisms

Three organisms were used in this study which included two strains of *C. albicans*: ATCC 10231 and ATCC 2091 and one strain of *C. auris*: NCPF 8971. The *C. albicans* strains were provided by the Southmead Hospital, Bristol, UK while the *C. auris* was purchased from the National Collection of Pathogenic Fungi (NCPF) operated by Public Health England (UK). The organisms were maintained on Sabouraud Dextrose agar (SDA, CM0041, Oxoid Ltd., UK) at 30 and 4 °C until used in tests. The inoculum was prepared from *C. albicans* and *C. auris* cultures, plated on SDA and incubated at 30 °C for 48 h and prior to each experiment, was standardized using 0.5 McFarland with a final concentration at approximately 10^6^ colony-forming units per millilitre (CFU/mL).

### Essential oils used in this study

The bark CEO (*Cinnamomum zeylanicum* Blume; CBEO) was donated by Amphora Aromatics Ltd., UK for research purposes and the leaf CEO (*Cinnamomum zeylanicum* Blume; CLEO) was purchased from Freshskin Beauty Ltd., UK. The EOs were kept at room temperature throughout the experimental period.

### Gas chromatography mass spectroscopy

Using similar methods to Zeng et al. (2015), gas chromatography mass spectroscopy (GC-MS) analysis was performed using the Agilent 6890 N Gas Chromatograph system (Agilent Technologies, USA) to identify the component profiles in the tested oils. Data acquisition and analysis was performed using the Agilent MassHunter software (Agilent Technologies, USA) based on the retention times and mass spectra.

### Antifungal susceptibility testing using the disc diffusion assay

#### Direct contact

The agar disc diffusion method described by Adukwu et al. (2016) was used for determination of the antifungal properties of CEOs. Miconazole (Sigma, USA) with the concentration at 10 μg/mL dissolved in DMSO (Sigma) was used as the reference antifungal drug. Briefly, 100 μL of yeast suspension was spread onto SDA plates and then paper discs (6 mm, Sigma) impregnated with 10 μL of essential oils or dissolved miconazole were placed over the agar surface, and plates were incubated at 30 °C for 48 h. Plates with discs in the absence of any treatment were used as reference controls. The diameter of the resulting inhibition zone in the fungal lawn was measured in millimetres.

#### Disc volatilisation assay

The antifungal activity of CEOs in vapour phase was investigated using the disc volatilisation technique (Boukhatem et al. 2014). Briefly, 100 μL of yeast suspension was spread onto SDA plates and then paper discs (6 mm, Sigma) impregnated with 10 μL of essential oils or dissolved miconazole were placed on the inside surface of the upper lid. The plate was sealed with parafilm to prevent leakage of the vapour and was promptly inverted on top of the lid. Plates were incubated at 30 °C for 48 h, and the diameter of the zone of inhibition was recorded in millimetres.

### Determination of minimum inhibitory concentration and minimum fungicidal concentration using broth dilution method

The method used in this study for determination of minimum inhibitory concentration (MIC) and minimum fungicidal concentration (MFC) of CEOs was adapted from Adukwu et al. (2012) with slight modifications. Briefly, Sabouraud Dextrose broth (SDB, Oxoid Ltd., UK) was prepared with a 0.5% (*v*/*v*) concentration of Tween 20 (Sigma, USA), used as the EO emulsifier. Both CBEO and CLEO were diluted twofold into SDB with Tween 20 to create a concentration range between 4 and 0.03% (*v*/*v*). The amount of 180 μL of the diluted CEOs was transferred into the corresponding wells of a 96-well microdilution plate with 20 μL of yeast suspension. Microplate wells of EO dilutions without yeast, and SDB and yeast suspension, were used as negative controls, and the plates incubated at 30 °C for 48 h. After the appropriate incubation time, the optical density (OD) of each well was recorded at 595 nm using the microplate reader (infinteF200Pro, Tecan, Switzerland). The MIC was defined as the lowest CEO concentration that produced inhibition of yeast growth.

MFC was measured by transferring 20 μL from each treated well of CEO concentration from 4% (*v*/*v*) to 0.03% (*v*/*v*) to labelled SDA plates followed by incubation at 30 °C for 48 h. The MFC was defined as the lowest CEO concentration that inhibited growth of the yeast or permitted less than three CFUs to occur, resulting thus in 99.9% fungicidal activity (Espinel-Ingroff et al. 2002).

### Effect of CEOs on the micromorphology of *C. albicans* and *C. auris*

To investigate changes in morphology caused by exposure to the CEOs against *C. albicans* and *C. auris*, we adapted the methods of Leite et al. (2014) with some modofications. One hundred microlitres (100 μL) of yeast suspension was spread onto cornmeal agar plates and then paper discs (6 mm, Sigma) were impregnated with 10 μL of essential oils and placed over the agar surface and the plates incubated at 30 °C for 48 h. Agar plates with discs without any treatments were used as reference controls. Plates were then visualised using the light microscope (Olympus, UK), tenfold magnification. Following incubation, two to three fungal colonies at the edge of the inhibitory zone were gently removed and placed on a clean microscope slide. A drop of Lacto-Fuchsin stain was placed on the sample and covered by a coverslip. The slides were examined under light microscope with 40-fold magnification.

### Modulation of cell membrane permeability

To assess the integrity of the yeast cell membranes following treatment with the CEO exposure, the cellular content leakage assay method described by Khan et al. (2013) was adapted with some modifications. Briefly, 200 μL of cell suspensions were transferred to wells of the 24-well plates (SLS, UK) containing 1800 μL of diluted CEOs at different concentrations (0.03, 0.015 and 0.008% (*v*/*v*) for CBEO; 0.13, 0.06 and 0.03% (*v*/*v*) for CLEO). CEOs in PBS served as blank, untreated cells in PBS as controls. Plates were incubated at 30 °C for 24 h. After the incubation period, the samples were centrifuged (1250 rpm, 2 min) and the release of cellular material in the supernatants was determined by using spectrophotometric measurement of cell supernatant at 260/280 nm (corresponding to nucleic acids and proteins).

### Scanning electron microscopy

Three sets of samples of the test strains were prepared with treatments at 0.03 and 0.015% (*v*/*v*) for CBEO and 0.13 and 0.06% (*v*/*v*) for CLEO. Control samples which consisted of the organisms without any treatment were also prepared. Fungal cultures were grown for 48 h in SDB, and 100 μL of the samples were dropped onto sterile metal discs and incubated for 48 h at 30 °C to establish fungal growth. The samples were then fixed using glutaraldehyde fixative. Periodic hourly rinsing of the samples three times with PBS was required to minimize any activity of the CEOs and to allow for clearing of any debris, which could affect the viewing of the samples. After that, samples were dehydrated using ethanol at different concentrations from 20 to 100% (*v*/*v*) and hexamethyldisilazane. Samples were then air dried, gold coated and visualized using the FEI Quanta FEG 650 SEM (Thermo Fisher Scientific, USA).

### Assessment of Haemolytic activity

#### Haemolytic activities of *Candida* strains and CEOs

To determine haemolytic activity of the *Candida* isolates, SDA medium containing 7% (*v*/*v*) horse blood (TCS Biosciences Ltd. UK) with or without 3% (*w*/*v*) glucose was used. A 10-μL yeast suspension or diluted CEOs at different concentrations from 4% (*v*/*v*) to 0.03% (*v*/*v*) was inoculated onto plates in triplicate; these were then incubated at 37 °C for 24 h in aerobic condition. After incubation, a transparent/semi-transparent zone around the inoculation site was considered positive haemolytic activity. The ratio of the diameter of the colony to that of the translucent zone of haemolysis (mm) was used as the haemolytic index, as described by Wan et al. (2015).

#### Effect of CEOs on the haemolytic activity of *Candida* isolates

The inhibition of haemolysin production in tested strains was conducted using the method of Manns et al. (1994) with some modifications. Briefly, *Candida* isolates were grown in SDB with 8% (*v*/*v*) glucose and CBEO at final concentrations of 0.03, 0.015 and 0.008% (*v*/*v*) and CLEO at final concentrations of 0.13, 0.06 and 0.03% (*v*/*v*) and incubated at 37 °C for 48 h. The SDB without CEOs was used as a control. The supernatant was collected from the incubated broth cultures by centrifuging at 5000 rpm for 30 min. Freshly washed sterile RBCs from horse blood (TCS Biosciences Ltd. UK) suspended in sterile PBS and enriched with 5% (*w*/*v*) glucose were mixed in a ratio of 1:1 with the supernatant and incubated at 37 °C for 3 h. Tubes were centrifuged at 2000 rpm for 10 min and read at 540 nm. Mean absorbance values were used to calculate the reduction percentage in the production of haemolysin in treated samples over untreated controls.

## Results

### Analysis of chemical composition (GC-MS)

The qualitative and quantitative composition of the used CBEO and CLEO is shown in Table 1. The number of identified components was greater in CLEO representing approximately 93% of the entire EO than the CBEO representing approximately 95%. The CLEO was characterized by high amounts of eugenol at 62.57% whilst in the CBEO this component was only 5.28%. The main component of CBEO was *trans*-cinnamaldehyde (66.43%) with <2% of this compound identified in the CLEO.

**Table 1:** GC-MS analysis of CBEO and CLEO showing major components

Components	Cinnamon leaf (%)	Cinnamon bark (%)
Bicyclo [3.1.1] hept-2-ene, 3,6,6-trimethyl-	2.54	–
Alpha-Phellandrene	2.27	–
Benzene, 1-methyl-4-(1-methylethyl)-	1.7	–
Cyclohexene, 1-methyl-5-(1-methylethenyl)-, (R)-	1.43	–
1,6-Octadien-3-ol, 3,7-dimethyl-	3.79	7.61
Eugenol	62.57	5.28
Caryophyllene	4.17	–
Benzyl benzoate	4.09	2.86
Phenol, 2-methoxy-4-(2-propenyl)-, acetate	3.49	–
*trans-*Cinnamaldehyde	1.92	66.43
1,3-Benzodioxole, 5-(2-propenyl)-	1.73	–
2-Propen-1-ol, 3-phenyl-, acetate	2.3	–
Eucalyptol	0.33	6.63
Butane, 2-methyl-	–	4.74
Benzaldehyde	0.27	1.04
Percentage of total components in the EO	92.6	94.59

### Antifungal susceptibility screening

Results from the antifungal susceptibility tests are summarized in Table 2. Both CBEO and CLEO demonstrated stronger antifungal activity against tested strains when compared with miconazole—an antifungal drug that is typically used in treatment of skin infections including those caused by yeast, e.g. candidiasis. The CBEO demonstrated greater inhibitory activity against *C. albicans* and *C. auris* in comparison with CLEO in the direct disk diffusion and disk volatilisation assays (Table 2). The CBEO was approximately twice as effective as the CLEO (direct contact) and approximately between 2 to 3.5 times more effective than CLEO (vapour assay).

**Table 2:** Inhibitory zones (mm) of fungal growth in *C. albicans* and *C. auris* by bark CEO, leaf CEO and miconazole (10 μg/mL) after 48 h of incubation

Selected yeasts	Mode of exposure or treatment	Bark CEO		Leaf CEO		Miconazole	
		Mean (mm)	SE	Mean (mm)	SE	Mean (mm)	SE
*C. albicans* ATCC 10231		53.00	1.68	26.32	0.67	24.33	1.37
*C. albicans* ATCC 2091	Direct contact	56.68	0.85	30.17	0.76	31.33	0.97
*C. auris* NCPF 8971		70.40	1.53	39.67	1.00	32.82	0.39
*C. albicans* ATCC 10231		55.52	0.65	15.08	0.82	NA	NA
*C. albicans* ATCC 2091	Vapour exposure	59.72	1.07	18.90	0.33	NA	NA
*C. auris* NCPF 8971		77.37	1.72	35.40	1.08	NA	NA

Results were from three independent experiments performed in triplicate (*N* = 3)

### Inhibitory and fungicidal activity

Results in Table 3 showed that *C. albicans* and *C. auris* were susceptible to CBEO and CLEO at low concentrations with both yeast species exhibiting greater susceptibility to CBEO with MIC and MFC activity observed at concentrations below 0.03% (*v*/*v*). The inhibitory action of CLEO against the *Candida* spp. at low concentrations between 0.06 and 0.13% (*v*/*v*) was effective at inhibiting the growth of *C. albicans* and *C. auris* strains; however, fungicidal activity of CLEO was observed at 0.25% (*v*/*v*).

**Table 3:** The antifungal activity of bark and leaf CEO (MICs and MFCs) against *C. albicans* and *C. auris*

Selected yeasts	Bark CEO		Leaf CEO	
	MIC % (*v*/*v*)	MFC % (*v*/*v*)	MIC % (*v*/*v*)	MFC % (*v*/*v*)
*C. albicans* ATCC 10231	< 0.03	< 0.03	0.13	0.25
*C. albicans* ATCC 2091	< 0.03	< 0.03	0.06	0.25
*C. auris* NCPF 8971	< 0.03	< 0.03	0.06	0.25

Results were from three independent experiments performed in triplicate (*N* = 3)

### Effect of CEOs on the micromorphology of *C. albicans* and *C. auris*

Results from the cornmeal agar assay showed that the *C. albicans* strains were predominantly comprised of filaments and after exposure to the EOs, hyphae formation was noticeably reduced in both *C. albicans* strains, especially *C. albicans* ATCC 2091. No change in morphology in the *C. auris* isolate was observed before and after exposure to both CEOs.

### Actions of CEOs on cell membrane permeability

**Fig. 1:**
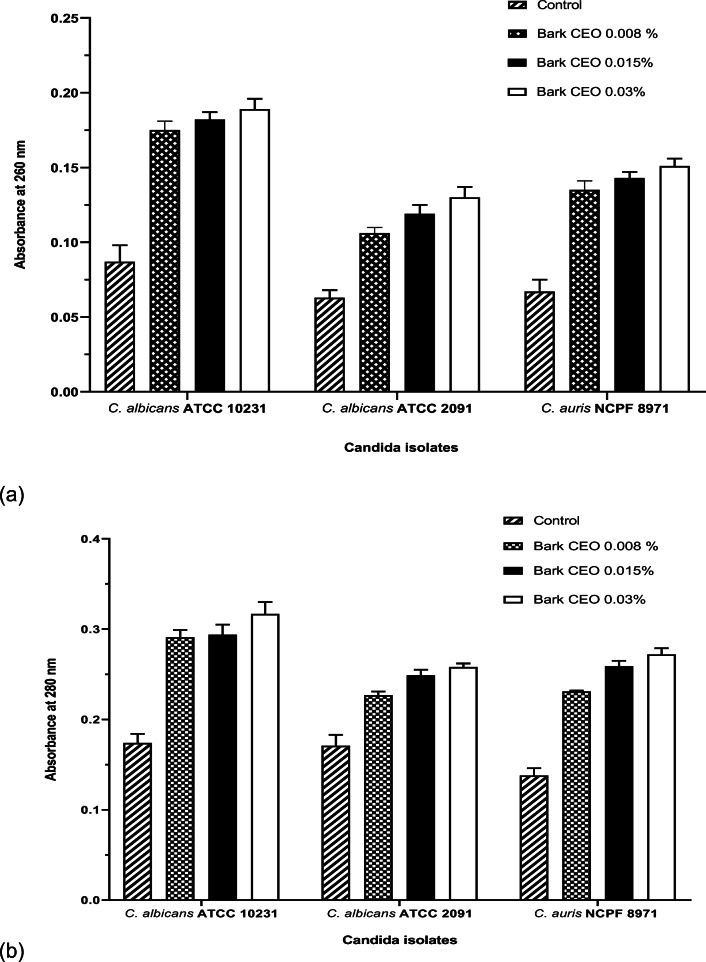
Assessment of (**a)** nucleic acid release, OD_260 nm_ and (**b)** protein release OD_280 nm_ from *C. albicans* and *C. auris* after treatment with cinnamon bark essential oil at 0.008, 0.015 and 0.03% (*v*/*v*). This experiment was carried out on three separate occasions (*N* = 3) with the bars showing mean values (± SE)

**Fig. 2:**
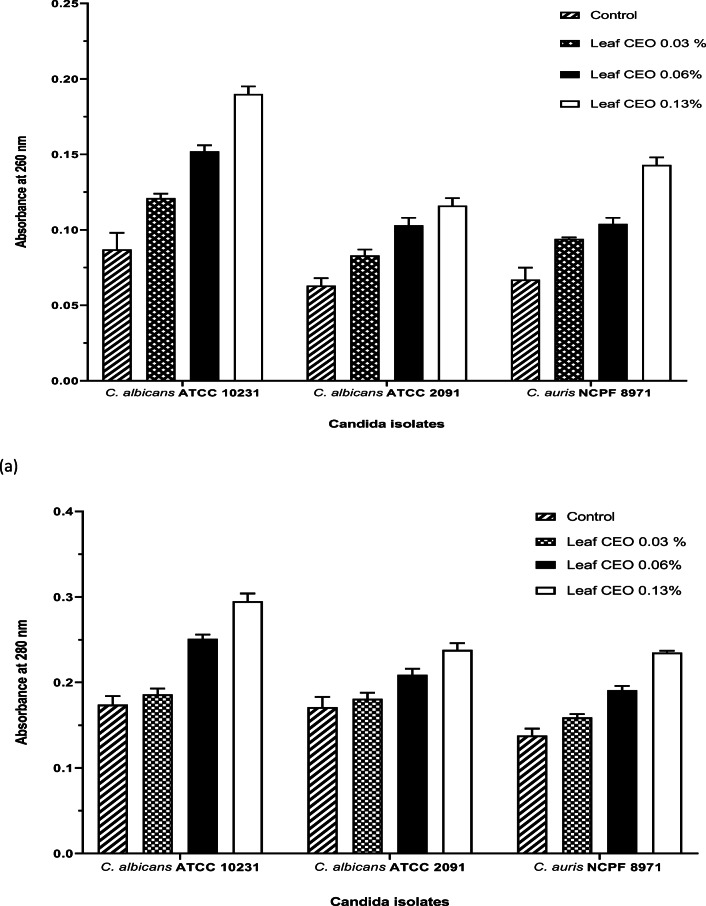
Assessment of (**a)** nucleic acid release, OD_260 nm_ and (**b)** protein release OD_280 nm_ from *C. albicans* and *C. auris* after treatment with cinnamon leaf essential oil at 0.03, 0.06 and 0.13% (*v*/*v*). This experiment was carried out on three separate occasions (*N* = 3) with the bars showing mean values (± SE)

**Fig. 3:**
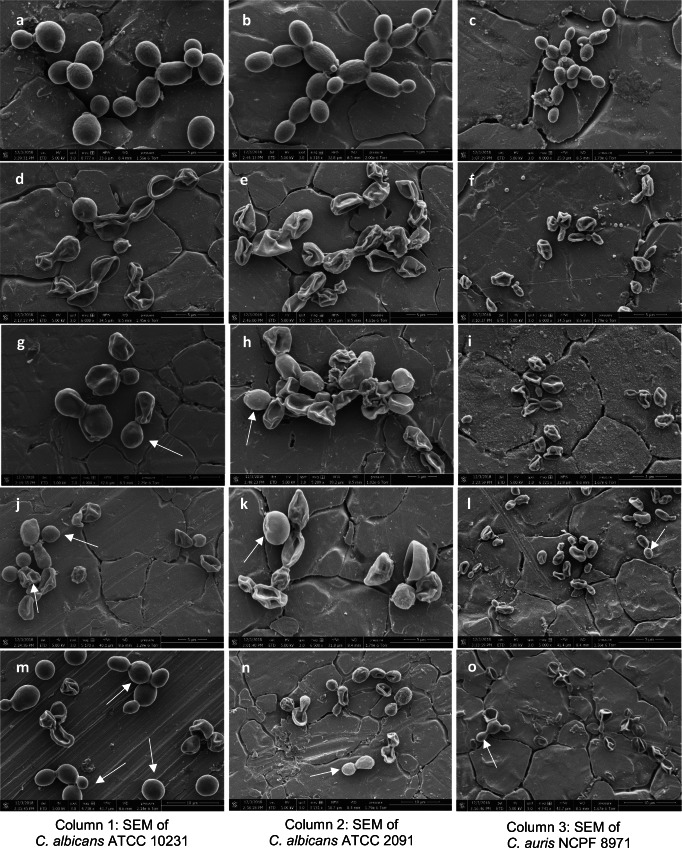
Scanning electron microphotographs of *C. albicans* ATCC 10231 (column 1), *C. albicans* ATCC 2091 (column 2) and *C. auris* NCPF 8971 (column 3). (**a**–**c)** Untreated cells; (**d**–**f)** Bark CEO 0.03% (*v*/*v*) treated cells; (**g**–**i)** Bark CEO 0.015% (*v*/*v*) treated cells; (**j**–**l)** leaf CEO 0.13% (*v*/*v* treated cells; (**m**–**o)** The white arrows indicate fungal cells which are undamaged or having a minor damage under treatments. Magnification, × 5000

The mechanism of action of CBEO and CLEO on *C. albicans* and *C. auris* was determined by cellular content leakage assay and scanning electron microscopy (SEM) morphological analysis following treatment with both CBEO and CLEO. The cellular content leakage assay shows that the envelop of the *Candida* spp. under investigation was damaged by the CEOs, demonstrated by the nucleic acid and protein release, i.e. cell lysis (Figs. 1 and 2). These results were supported by SEM images, which revealed receding of the cell membrane and shrinkage of cell surfaces (Fig. 3).

In the cell content leakage assay, an increase in OD_260 nm_ indicates a release of nucleic acid whilst the OD_280 nm_ shows protein release. Following exposure of the test organisms to CBEO, nucleic acid and protein release were observed (*p* < 0.05). In addition, the higher the concentration, the greater the nucleic acid and protein leakage. Figure 2 also demonstrates cell content leakage following exposure to CLEO at the MIC and concentrations below the MIC; however, the cell content leakage was not significantly different (*p* > 0.05) from the untreated samples at 0.06 and 0.03% (*v*/*v*).

The MIC and MFC of CBEO were found to be below the test concentration range < 0.03% (*v*/*v*) and at 0.015% (*v*/*v*); the three test organisms were damaged and had lost turgidity and integrity when exposed to these concentrations (observed by SEM). *C. albicans* ATCC 2091 and *C. auris* NCPF 8971 were killed at 0.13% (*v*/*v*) of CLEO while the control cells appeared turgid and whole with smooth cell membrane (Fig. 3a–c). Figure 3 (m-o) shows some undamaged cells when the cells were exposed to CLEO at 0.06% (*v*/*v*; MIC for *C. albicans* ATCC 2091 and *C. auris* NCPF 8971) which is a confirmation of fungistatic activity at that concentration and at 0.13% (*v*/*v*), the cells were damaged by the CLEO.

### Haemolysin activities of *Candida* isolates

In the glucose-enriched agar plates, two different types of haemolysis were observed circumscribing the yeast “colony” when viewed with transmitted light. The first was a totally translucent ring identical to beta-haemolysis, and the second was a greenish-black halo comparable with alpha-haemolysis. At 24 h incubation, only alpha-haemolysis was observed in the glucose-free plates. However, after 48 h incubation, only beta-rings were observed in glucose-free plates. We clearly observed greater haemolytic activity in the three tested strains in the glucose-enriched plates compared with the glucose-free plates.

Results in Table 4 show the haemolysin activities of *Candida* isolates. In both glucose-enriched and glucose-free plates, haemolysis activity was higher in *C. albicans* ATCC 10231 with the mean diameters of alpha- and beta-rings in glucose-enriched plates after 24 h incubation being 15.51 ± 0.36 and 21.46 ± 0.6 mm, respectively. Meanwhile, in the glucose-free agar plates, the mean diameter of alpha-rings of *C. albicans* ATCC 10231 was 11.42 ± 0.31 mm. After 48 h incubation, the mean diameters of alpha- and beta-rings of *C. albicans* ATCC 10231 in glucose-added plates were still the highest among three tested stains. The mean diameter of beta-rings of *C. albicans* ATCC 10231 in glucose-free plates was 16.63 ± 0.41 mm. In contrast, haemolysis was lower in *C. auris* NCPF 8971 with mean alpha- and beta-rings in glucose-enriched plates after 24 h incubation were 10.61 ± 0.4 and 16.43 ± 0.43 mm, respectively. After 48 h incubation, the mean diameter of beta-rings of *C. auris* NCPF 8971 in glucose-free plates was 14.56 ± 0.23 mm.

**Table 4:** Diameters of α- and β-rings (mm) representing haemolytic activity of *C. albicans* ATCC 10231, *C. albicans* ATCC 2091 and *C. auris* NCPF 8971 with and without glucose enrichment after incubation for 24 and 48 h

Organisms	Glucose conc. (*w*/*v*)	Incubation time (h)	α-Ring (mm)	β-Ring (mm)
*Candida albicans* ATCC 10231	3	24	15.51 (± 0.36)	21.46 (± 0.60)
	0		11.42 (± 0.31)	0 (non-haemolytic)
	3	48	17.72 (± 0.15)	28.83 (± 0.46)
	0		0 (non-haemolytic)	16.63 (± 0.41)
*Candida albicans* ATCC 2091	3	24	14.26 (± 0.33)	20.7 (± 0.43)
	0		10.96 (± 0.27)	0 (non-haemolytic)
	3	48	17.7 (± 0.35)	26.2 (± 0.75)
	0		0 (non-haemolytic)	16.38 (± 0.37)
*Candida auris* NCPF 8971	3	24	10.61 (± 0.40)	16.43 (± 0.43)
	0		9.7 (± 0.16)	0 (non-haemolytic)
	3	48	15.11 (± 0.36)	24.54 (± 0.43)
	0		0 (non-haemolytic)	14.56 (± 0.23)

Results were from three independent experiments performed in triplicate (*N* = 3)

### Effects of bark and leaf CEOs on the haemolytic activity of *C. albicans* and *C. auris*

**Fig. 4:**
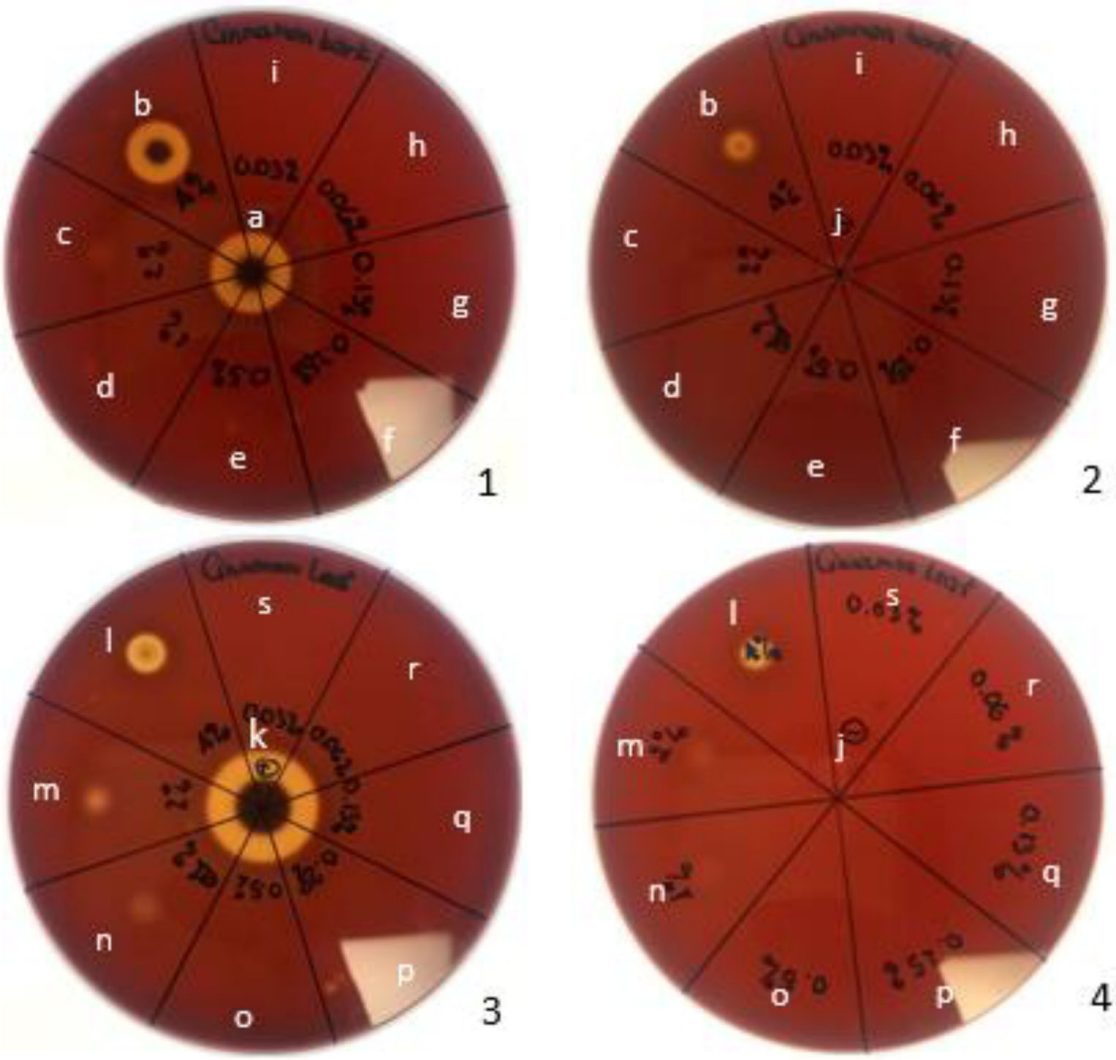
Photographs depicting the hemolysis of horse blood agar (supplemented with 3% w/v of glucose) induced by different concentrations of bark CEO (1, 2) and leaf CEO (3, 4). Panel a shows pure bark CEO (a) and pure leaf CEO (k) served as positive control; Sabouraud dextrose broth +0.5% Tween 20 served as negative control (j); bark CEO 4% (b); bark CEO 2% (c); bark CEO 1% (d); bark CEO 0.5% (e); bark CEO 0.25% (f); bark CEO 0.125% (g); bark CEO 0.06% (h); bark 0.03% (i); leaf CEO 4% (l); leaf CEO 2% (m); leaf CEO 1% (n); leaf CEO 0.5% (o); leaf CEO 0.25% (p); leaf CEO 0.125% (q); leaf CEO 0.06% (r); and leaf CEO 0.03% (s)

An antifungal agent may either increase or attenuate the virulence factors released by fungal cells (Khan and Ahmad, 2013). Clinical performance of an antifungal compound is not only determined by its fungicidal or fungistatic activity but also by its influence on virulence factor release (Lopes et al. 2013). To ensure that the CEO concentration in this test was not directly responsible for the haemolytic activity following the challenge with the yeast species under investigation, assays to test the haemolysis activity of CBEO and CLEO were conducted on horse blood agar (Fig. 4). The results indicated that both CEOs could cause haemolytic activity at high concentration; however, at concentrations ≤1% (*v*/*v*) for CBEO and < 0.5% (*v*/*v*) for CLEO, both CEOs did not cause any haemolytic activity in vitro.

**Fig. 5:**
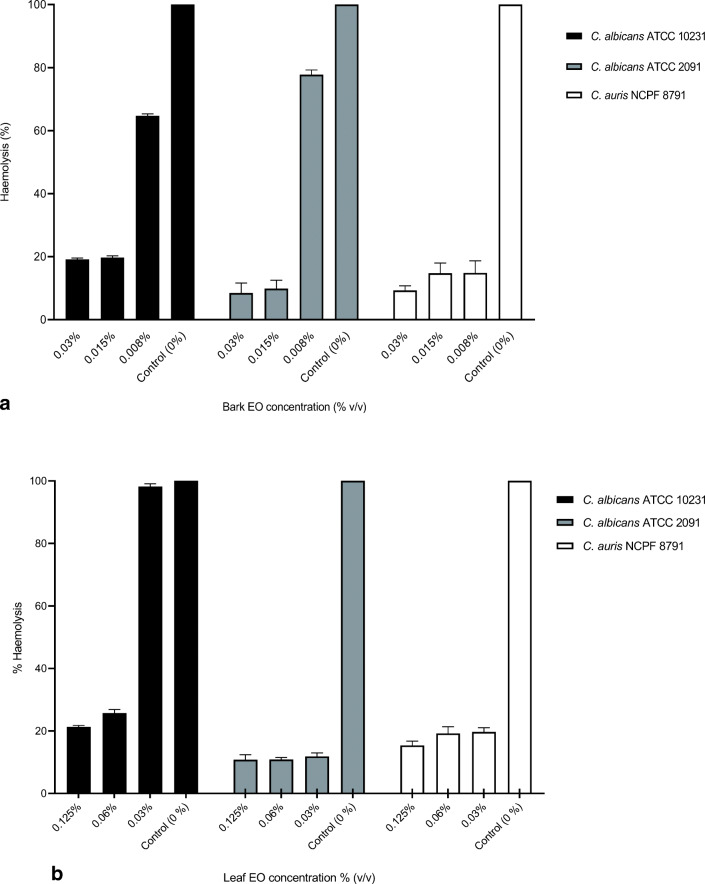
Effects of CBEO (**a**) and CLEO (**b**) at different concentrations on the inhibition of haemolysis in *C. albicans* ATCC 10231; *C. albicans* ATCC 2091 and *C. auris* NCPF 8971. This experiment was carried out on three separate occasions and in triplicate (*N* = 3) with the bars showing mean values (± SE)

The results from the inhibitory effects of CBEO and CLEO on the haemolytic activity of *C. albicans* and *C. auris* are presented in Fig. 5. Following treatment with both CEOs at different concentrations, haemolytic activity was reduced in all tested organisms. CBEO at 0.03% (*v*/*v*) and 0.015% (*v*/*v*) reduced haemolysis in the tested strains to lower than 20% overall and at 0.008% (*v*/*v*) haemolysis was reduced by approximately 30% (Fig. 5a). At the same concentration, haemolysis was reduced by over 80%. CLEO also reduced haemolytic activity of *C. albicans* and *C. auris* at the MIC and the concentration 2× MIC (Fig. 5b). At the sub-inhibitory concentration, 0.5× MIC, there was no significant difference between the treated and untreated strain (*C. albicans* ATCC 10231; *p* > 0.05).

## Discussion

### Analysis of chemical composition (GC-MS)

The GC-MS results in this study were similar to that of Mayaud et al. (2008) with the exception of cinnamaldehyde, which was not found in the CLEO, analysed in their paper. Previous studies have shown that both *trans*-cinnamaldehyde and eugenol, the key components of CBEO and CLEO, respectively have been are antimicrobial against bacteria and fungi. In a recent review article by Doyle and Stephens (2019), *trans*-cinnamaldehyde was found to be responsible for causing damage to the bacterial cells via different methods; cell release activity, anti-quorum sensing activity, effect on ATP etc. Eugenol, on the other hand, has been shown to cause morphological changes and damage to *Candida albicans*-adherent cells and biofilms (He et al. 2007). To our knowledge, very little is known on the effects of EOs against the new pathogen *C. auris* and there are no published reports on the effects of CEOs against this pathogen.

### Antifungal activity of CBEO and CLEO

The susceptibility of *C. albicans* and *C. auris* to the antifungal effects of bark and leaf CEO when in contact with the fungal lawns and in vapour phase was determined. The results were consistent with the studies by Brnawi et al. (2018) and Ranasinghe et al. (2002) who found CBEO to be a more effective antimicrobial than CLEO. Research into the antimicrobial vapour investigations using EOs is gaining interest, and our results further support this with CBEO demonstrating strong antimicrobial activity in vapour phase while CLEO produces major inhibition in direct contact. The main component of CBEO is *trans*-cinnamaldehyde and that of CLEO is eugenol. The study by Sanla-Ead et al. (2012) investigated the antifungal activity of *trans*-cinnamaldehyde and eugenol and found that *trans*-cinnamaldehyde displayed stronger antifungal activity in vapour phase which might explain why CBEO was more effective in vapour phase when compared with CLEO. Antimicrobial activity via vapour diffusion offers a different delivery mode for the CEOs and other essential oils for the treatment and management of infections, e.g. in clinical settings to improve air quality by means of decontamination.

Both CBEO and CLEO demonstrated fungicidal properties at very low concentrations. *C. auris* has been described as a notorious nosocomial pathogen requiring urgent efforts to understand and to identify therapeutic options due to high transmissibility, challenges with identification, incorrect use of antifungal drugs and treatment failures, which lead to high mortality (Sears and Schwartz 2017; Sardi et al. 2018). The limited antifungal treatment options for this important pathogen creates a need to develop new alternative treatments in line with the global antimicrobial resistance strategies, which indicates the development of new treatments and reduce reliance on existing conventional antimicrobials as an important step.

### Effect of CEOs on the micromorphology of *C. albicans* and *C. auris*

Germination is considered one of the major virulence factors known to contribute to *Candida* pathogenesis (Larkin et al. 2017). In *C. albicans*, virulence gene expression is linked to its ability to transition from yeast form to hyphal form (Gow et al. 2002). The yeast-to-hyphal transition in *C. albicans* is known to promote virulence because hyphae can exert mechanical force to breach and damage endothelial cells (Thompson et al. 2011). Moreover, macrophages and neutrophils can be lysed by the growth of *C. albicans* hyphae (Lo et al. 1997). In the presence of CEOs, hyphal formation was inhibited in the *C. albicans* strain ATCC 2091, suggesting a potential mechanism of action of the EO as hyphae formation is associated with release of virulence factors. The findings also showed that under similar test conditions, *C. auris* NCPF 8971 did not germinate, form hyphae or produce chlamydospores. Since the ability of *Candida* to invade endothelial cells is important in causing candidemia and candidiasis, the inability to form hyphae of *C. auris* NCPF 8971 suggests that virulence as described in other studies is likely as a result of other factors and requires further investigation.

### Actions of CEOs on cell membrane permeability

The mechanism of action of CBEO and CLEO on *C. albicans* and *C. auris* was damage to the envelop, evident by the shrinkage of the cell surfaces and receding of cytoplasm leading to lysis of cells. In addition, the level of membrane damages of *Candida* cells correlated with the concentration of CEOs used. This finding suggests that the membrane-destructive action of both CBEO and CLEO depends on the concentration of oils. This conclusion was re-confirmed by the increase in the level of nucleotide and protein in the extracellular media. Khan et al. (2013) proposed that membranous structures such as cell wall and cell membrane of fungal cells are the target sites of *trans*-cinnamaldehyde and eugenol. This activity, in part, can be due to lipophilic properties of these compounds for their partition into the lipid bilayer of the plasma membrane (Knobloch et al. 1989), leading to the disruption of the structure of different membrane layers and subsequently permeability. Another study by Shahina et al. (2018) using utilizing atomic force microscopy (AFM) imaging, laser scanning confocal Microscopy (LSCM) imaging and Congo red staining techniques, demonstrated cell surface exfoliation, altered ultrastructure, delayed cell cycle and reduced cell wall integrity in *C. albicans* cells following exposure to CBEO. In their study, the findings suggested that not only cinnamaldehyde possess the ability to compromise cell membrane and wall integrity but also minor components of CBEO such as limonene, eugenyl acetate, linalool and benzyl benzoate contribute to CBEO antifungal activity by other routes of impact such as introducing spindle defect or arresting cell cycle in the anaphase. Further assays are needed to determine other sites of action of the CEOs on *Candida* spp., e.g. transmission electron microscopy, sorbitol protection assay or flowcytometry. However, the results from this study clearly demonstrate antifungal activity of both CBEO and CLEO against the *C. albicans* and for the first time, against *C. auris*. Both species of *Candida* irrespective of morphology switching action had significantly damaged cell membrane structures when exposed to low concentrations of the EOs.

### Antihaemolytic effect of CEOs

To date, very few studies have investigated the haemolytic activity of *C. auris* although it has been shown to be a great source of nosocomial bloodstream infections (Sardi et al. 2018). Haemolytic activity has been demonstrated in several studies and is a factor in the pathogenesis of *C. albicans* (Luo et al. 2001; Favero et al. 2014). In our study, both *C. albicans* and *C. auris* produced haemolysis in the glucose-enriched blood media, which is of importance as haemolysis activity in the presence of glucose has been indicated as a possible factor in the pathogenesis of *Candida* in diabetic patients (Malcok et al. 2009). However, with the presence of CEOs, at MIC and sub-MICs, both CBEO and CLEO inhibited production of haemolysin factors in *C. albicans* and *C. auris* which offers some insight into a not well-described activity of EOs on *Candida* spp. supporting our initial findings from the cornmeal agar assay where switching to hyphal form was inhibited in the presence of the CEOs. In the review article by Nayak et al. (2013), there is enhanced haemolytic activity in *C. albicans* which is characterized by iron uptake leading to and secretion of haemolysin, facilitating hyphal invasion during candidiasis infections. Thus, the reduction of haemolytic activity, the prevention of morphology switching from spore to hyphae and damage to the membrane are demonstrated here as some of the mechanisms of action of the cinnamon EOs against the *Candida* species. The potential of cinnamon EO as an antifungal agent is also evident with the inhibitory and fungicidal action against *C. albicans* and *C. auris* at low concentrations of both leaf and bark oils. Both EOs were shown to be fast-acting antimicrobials against *Pseudomonas aeruginosa* in a recent study by Elcocks et al. (2020), and with such promising action against two troublesome pathogenic *Candida* species, there is a clear need to explore this EO further as a treatment option for candidiasis infections.

In summary, the findings reported here demonstrate for the first time the potential of both bark and leaf CEOs to exert antifungal activity against *C. auris*, which is a newly found *Candida* spp., along with *C. albicans*. Mode of action of these oils towards fungal cells determined by SEM analysis, highlight their ability to damage the membranous structures of fungal cells. In this study, test *Candida* strains and CEOs at high concentrations all showed haemolytic activities. At MIC and sub-MICs, both bark and leaf CEOs reduced the germination and haemolysin factors in *C. albicans* and *C. auris*. Further in vivo studies are needed to assess any potentially toxic effect of CEOs; however, the antifungal and anti-haemolytic activity raise another interesting field in researching antifungal therapeutic interventions in both clinical and industrial applications.

## Data Availability

Not applicable.
